# Aneuploidy is frequent in heterozygous diploid and triploid hydatidiform moles

**DOI:** 10.1038/s41598-024-57465-5

**Published:** 2024-03-22

**Authors:** P. Walbum, L. Andreasen, M. Geilswijk, I. Niemann, L. Sunde

**Affiliations:** 1 Department of Clinical Genetics, Aalborg University Hospital, Aalborg, Denmark; 2 Department of Clinical Genetics, Aarhus University Hospital, Aarhus, Denmark; 3 Department of Gynecology and Obstetrics, Randers Regional Hospital, Randers, Denmark

**Keywords:** Cytogenetics, Genotyping and haplotyping, Chromosome segregation, Cytogenetics, Genotype, Aneuploidy, Uniparental disomy

## Abstract

Hydatidiform moles are abnormal conceptuses. Many hydatidiform moles are diploid androgenetic, and of these, most are homozygous in all loci. Additionally, most hydatidiform moles are euploid. Using Single Nucleotide Polymorphism (SNP) array analysis, in two studies a higher frequency of aneuploidy was observed in diploid androgenetic heterozygous conceptuses, than in their homozygous counterparts. In the Danish Mole Project, we analyze conceptuses suspected to be hydatidiform moles due to the clinical presentation, using karyotyping and Short Tandem Repeat (STR) analysis. Among 278 diploid androgenetic conceptuses, 226 were homozygous in all loci and 52 (18.7%) were heterozygous in several loci. Among 142 triploid diandric conceptuses, 141 were heterozygous for paternally inherited alleles in several loci. Here we show that the frequencies of aneuploidy in diploid androgenetic heterozygous and triploid diandric heterozygous conceptuses were significantly higher than the frequency of aneuploidy in diploid androgenetic homozygous conceptuses. In diploid androgenetic and triploid diandric conceptuses that are heterozygous for paternally inherited alleles, the two paternally inherited sets of genomes originate in two spermatozoa. Each spermatozoon provides one pair of centrioles to the zygote. The presence of two pairs of centrioles may cause an increased risk of aneuploidy.

## Introduction

A hydatidiform mole (HM) is an abnormal conceptus, and in most HMs the genome is diploid androgenetic or triploid diandric^1^. In most diploid androgenetic conceptuses, homozygosity is found in every locus (androgenetic homozygous diploids), whereas in some diploid androgenetic conceptuses, heterozygosity is found in some loci (androgenetic heterozygous diploids)^2^. Conversely, in some triploid diandric conceptuses, the two paternally inherited sets of genomes are identical (diandric homozygous triploids), whereas in most triploid diandric conceptuses, two non-identical paternally inherited alleles are observed in several loci (diandric heterozygous triploids)^3^.

Aneuploidy has been observed in diploid androgenetic conceptuses^4,5^ and in triploid diandric conceptuses^3,6^. Using DNA marker analyses, Usui et al. and Finley et al. observed a higher frequency of aneuploidy among 31 and 19 diploid androgenetic heterozygous conceptuses than among 238 and 177 diploid androgenetic homozygous conceptuses, respectively^5,7^.

In this paper, we report frequencies of aneuploidy in diploid androgenetic and triploid diandric conceptuses, determined by karyotyping. We observed higher frequencies of aneuploidy both in diploid androgenetic heterozygous conceptuses and triploid diandric heterozygous conceptuses, than in diploid androgenetic homozygous conceptuses.

## Methods and material

In the Danish Mole Project, we collect fresh samples of evacuated tissue from conceptuses suspected by the gynecologist to be HMs due to observations by ultrasound, vesicular villi observed in the evacuated tissue, and/or high levels of human chorionic gonadotropin (hCG). Samples are received from hospitals in Western Denmark (approx. one million inhabitants)^8^.

### Karyotyping

Uncultured and/or cultured cells were karyotyped. For uncultured cells, the instruction was to count the chromosomes in five metaphases and perform karyotyping of at least one metaphase. For cultured cells, the instruction was to count the chromosomes in 10 metaphases and perform karyotyping of at least one metaphase. If the number of chromosomes and the sex chromosomes in all metaphases matched the karyotype in the metaphase that was karyotyped, all metaphases were assumed to have the same karyotype. If an abnormity was observed in one metaphase, karyotyping of all metaphases was performed. As maternal cells may have been present, we disregarded metaphases with the karyotype 46,XX in cases where other metaphases showed a Y-chromosome and/or were polyploid, and genotyping identified no sign of mosaicism. We also disregarded metaphases with karyotypes, which were not seen in at least two other metaphases, as these karyotypes were potentially caused by artefacts.

We classified a conceptus as successfully karyotyped if the same karyotype was observed in at least three metaphases. We classified a conceptus as aneuploid if the same aneuploid karyotype was observed in at least three metaphases. We classified a conceptus as diploid if the modal number of chromosomes was 45–48. We classified a conceptus as triploid if the modal number of chromosomes was 66–77. If two different karyotypes were observed, each in at least three metaphases, the conceptus was classified as mosaic.

### Molecular genotyping

DNA was extracted from fresh frozen chorionic villi, and electrophoresis was performed as previously described^9,10^. The parental origin of the genome in a conceptus was estimated by comparing the results of Short Tandem Repeat (STR) analysis for the conceptus, with the results for the patient, or for both the patient and her partner. We used the AmpFLSTR Identifiler kit (Applied Biosystems) or the AmpFLSTR NGM SElect (Applied Biosystems) kit. AmpFLSTR Identifiler (Applied Biosystems) covers Amelogenin and the following 15 unlinked polymorphic loci: D8S1179, D21S11, D7S820, CSF1PO, D3S1358, TH01, D13S317, D16S539, D2S1338, D5S818, FGA, D19S433, vWA, TPOX, D18S51. AmpFLSTR NGM SElect (Applied Biosystems) covers Amelogenin and the following 16 unlinked polymorphic loci: D3S1358, vWA, D16S539, D2S1338, D8S1179, D21S11, D18S51, D19S433, TH01, FGA, D10S1248, D22S1045, D2S441, D1S1656, D12S391, SE33. We classified an analysis as successful if results were obtained for at least nine unlinked loci.

We classified diploid androgenetic conceptuses and triploid diandric conceptuses as heterozygous or homozygous, as described in Table 1.

**Table 1:** Classification of diploid androgenetic heterozygous conceptuses, diploid androgenetic homozygous conceptuses, triploid diandric heterozygous conceptuses, and triploid diandric homozygous conceptuses.

	Diploid androgenetic^a^	Triploid diandric^a^
Heterozygous	In all loci, peak(s) with height(s) consistent with diploidy were observed, heterozygosity was observed in at least three loci, and in at least three loci no allele was identical with a maternal allele	In all loci, peak(s) with height(s) consistent with triploidy were observed, in all loci one allele was identical with a maternal allele, and in at least three loci two alleles were not identical with a maternal allele
Homozygous	In all loci homozygosity was observed, and in at least three loci the allele was not identical with a maternal allele	In all loci, at maximum two peaks with height(s) consistent with triploidy were observed. In all loci one allele was identical with a maternal allele, and in at least 3 loci the other allele was not identical with a maternal allele and the height of the peak suggesting two paternally inherited sets of genomes

^a^In conceptuses where aneuploidy was observed, the findings in some loci could deviate in accordance with the aneuploidy, see also Fig. 2.

**Figure 1:**
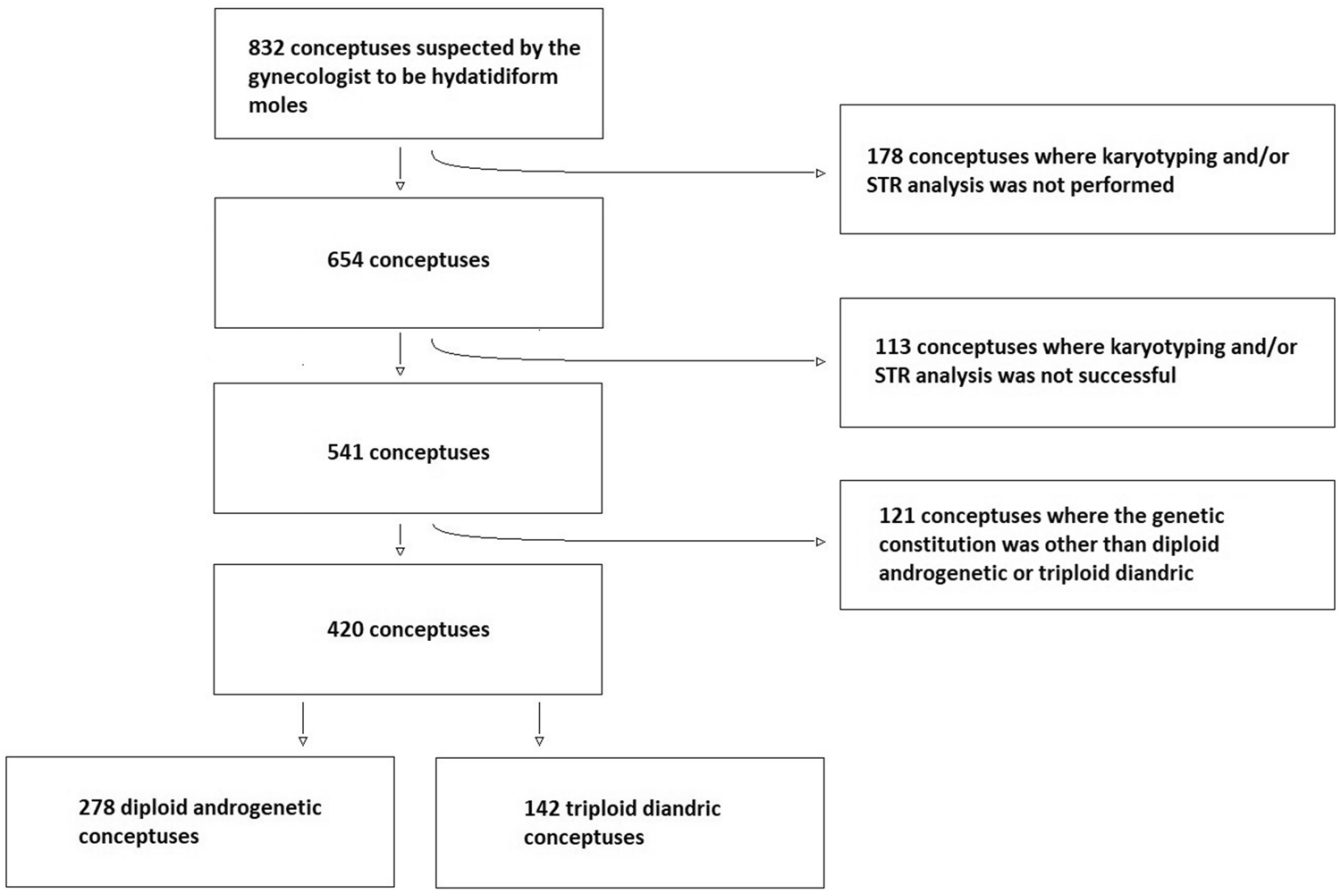
Flowchart for including conceptuses.

We included samples received between April 1st 1986 and December 31st 2021, where chorionic villi were observed, where karyotyping was successful and diploidy or triploidy was observed, and where analysis of the parental origin of the genome was successful and two paternally inherited sets of genomes were observed (Fig. 1).

Some observations in triploid conceptuses have been published previously^6^.

### Data analyses

Data was handled with Stata (Stata/MP 17.0, StataCorp, College Station, TX, USA). Results were evaluated by Fisher’s exact test and Welch’s unequeal variances t-test.

### Ethics

The project was registered in the Region of Northen Jutland according to the General Data Protection Regulation, article 30 (ID-number: F2023-021). The Danish Mole Project was approved by The Danish National Committee of Ethics in Science (1-10-72-370-13). All methods were performed in accordance with the relevant guidelines and regulations. Informed consent was obtained from all participants.

## Results

278 conceptuses were classified as diploid and androgenetic, whereas 142 conceptuses were classified as triploid and diandric (Fig. 1).

**Figure 2:**
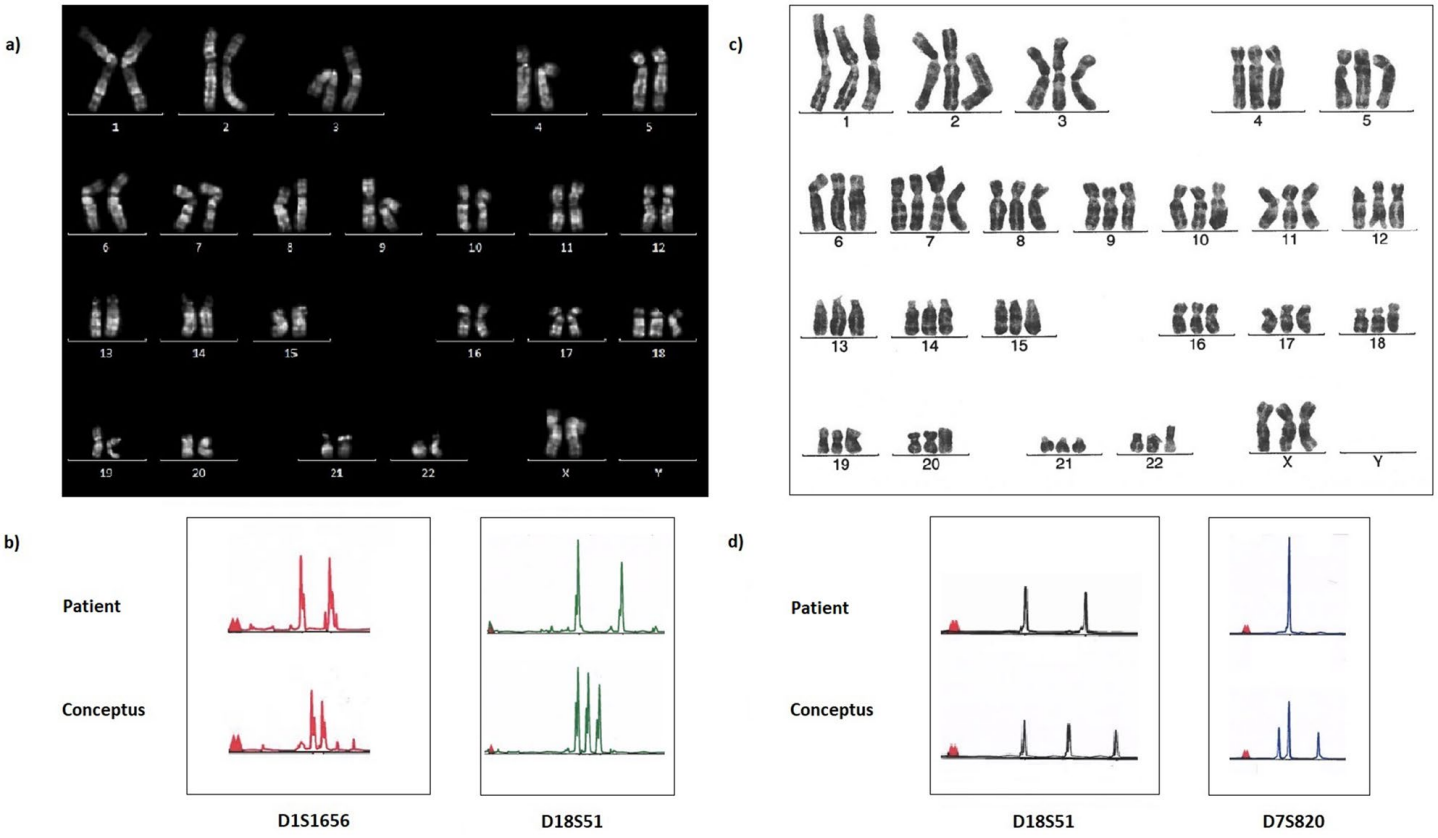
Karyotype and partial electropherograms for two conceptuses with aneuploidy. (**a**,**b**) Conceptus 1023, diploid androgenetic. (**a**) Q-banded karyotype showing diploidy, except for trisomy 18. (**b**) For locus D1S1656 (1q42), the electropherogram for the conceptus show two different peaks of similar heights, none are identical with a peak in the electropherogram for the patient. Similar results were observed in other loci. Together with the observation of a diploid karyotype, this indicates that the conceptus was diploid androgenetic heterozygous. Consistently with the observation of trisomy 18 by karyotyping, three peaks were observed for locus D18S51 (18q22.33). By comparing with the electropherogram for the patient, it is seen that the two alleles were paternally inherited, and one allele maternally inherited, indicating that the extra chromosome 18 was maternally inherited. (**c**,**d**) Conceptus 698, triploid diandric. (**c**) G-banded karyotype showing triploidy, except for tetrasomy 7. (**d**) For locus D18S51 (18q22.33), the electropherogram for the conceptus show three different peaks of similar heights, of which two are not identical with a peak in the electropherogram for the patient. Similar results were observed in other loci. Together with the observation of a triploid karyotype, this indicates that the conceptus was triploid diandric and heterozygous for the paternally inherited alleles. Consistently with the observation of tetrasomy 7 by karyotyping, the electropherogram for the conceptus show three peaks for locus D7S820 (7q21.11), of which one has approximately double height. By comparing with the electropherogram for the patient, it is seen that two alleles were paternally inherited, and one allele maternally inherited. The double height of the peak identical with a peak in the electropherogram for the patient, indicate that the extra chromosome 7 was maternally inherited.

For each conceptus of 194 diploid androgenetic conceptuses, the same karyotype was observed in 10 metaphases or more, whereas the same karyotype was observed in 3–9 metaphases in 84 conceptuses. In 263 diploid conceptuses, the STR analysis was successful for at least 16 loci, and in 15 conceptuses 9–15 loci were successfully analyzed. Among the 278 diploid androgenetic conceptuses, 226 were classified as homozygous for the paternally inherited sets of genomes, whereas 52 (18.7%) were classified as heterozygous. Aneuploidy was observed in seven diploid conceptuses (2.5%) (Table 2). In some cases, the aneuploidy detected by karyotyping, was also detectable by genotyping, one example is illustrated in Fig. 2.

**Table 2:** Parental origin of the genome and karyotype in seven of 278 diploid androgenetic conceptuses in which aneuploidy was observed.

Conceptus	Parental origin of the genome^a^	Karyotype^b^
209	P1P1	mos 47,XX, + 20[5]/46,XX[13]
416	P1P2	45,X[10]
695	P1P2	47,XY, + 8[15]
699	P1P2	mos 48,XX, + 3, + 9[10]/46,XX[3]
841	P1P2/PM	mos 48,XY, + 7, + 18[12]/46,XY[6]
976	P1P2	mos 47,XXY[10]/46,XY[4]
1023	P1P2	47,XX, + 18[9]

^a^P1P1: Diploid androgenetic homozygous conceptus. P1P2: Diploid androgenetic heterozygous conceptus. P1P2/PM: By karyotyping, the conceptus was classified as diploid, in three loci three alleles were observed, of which only one was identical to a maternal allele, and in all loci the height of the peaks indicated that two diploid cell lines were present, one androgenetic and one biparental^10^.

^b^In squared brackets are given the number of metaphases where the karyotype indicated was observed. The term “mos” is used where cells with two different karyotypes were observed, although we have not specifically analyzed whether this was caused by mosaicism or chimerism.

The frequency of aneuploidy in diploid androgenetic heterozygous conceptuses was significantly higher than the frequency of aneuploidy in diploid androgenetic homozygous conceptuses, 11.5% and 0.4%, respectively (p = 0.0002, Fisher’s exact test).

In each conceptus of 53 triploid diandric conceptuses, the same karyotype was observed in 10 metaphases or more, whereas the same karyotype was observed in 3–9 metaphases in 89 conceptuses. In 135 triploid conceptuses, the STR analysis was successful for at least 16 loci, and in seven conceptuses 9–15 loci were successfully analyzed. Among 142 diandric triploid conceptuses, 141 were classified as heterozygous for the paternally inherited sets of genomes, whereas one was classified as homozygous. Aneuploidy was observed in 21 conceptuses (14.8%) (Table 3). In some cases, the aneuploidy detected by karyotyping, was also detectable by genotyping (Fig. 2).

**Table 3:** Parental origin of the genome and karyotype in 21 of 142 triploid diandric conceptuses in which aneuploidy was observed.

Conceptus	Parental origin of the genome^a^	Karyotype^b^
297	P1P2M	mos 70,XXX, + 9[3]/69,XXX[4]
426	P1P2M	70,XXY, + 6[7]
469	P1P2M	70,XXY, + 14[7]
506	P1P2M	mos 68,XXX,− 11[5]/69,XXX[7]
533	P1P2M	73,XXX, + 3, + 4, + 15, + mar[3]
538	P1P2M	70,XXX, + 20[11]
615	P1P2M	70,XXXY[9]
633	P1P2M	70,XXX, + 7[6]
664	P1P2M	68,XXY,− 11[12]
668	P1P2M	70,XXX, + 6[6]
698	P1P2M	70,XXX, + 7[7]
814	P1P2M	68,XX[6]
909	P1P2M	71,XXY, + 3, + 21[3]
914	P1P2M	68,XXY,− 13[13]
941	P1P2M	77,XXXX, + 2, + 3, + 7, + 8, + 8, + 13, + 20[10]
943	P1P2M	mos 69,XX, + 17[4]/70,XXX, + 17[10]
986	P1P2M	68,XY[7]
1070	P1P2M	71,XXXX, + 17[11]
1059	P1P2M	70,XXY, + 13[15]
1218	P1P2M	70,XXY, + 7[14]
1338	P1P1M	mos 68,XXX,− 20[5]/69,XXX,− 20, + mar[9]

^a^P1P1M: Triploid diandric homozygous conceptus. P1P2M: Triploid diandric heterozygous conceptus.

^b^In squared brackets are given the number of metaphases where the karyotype indicated were observed. The term “mos” is used where cells with two different karyotypes were observed, although we have not specifically analyzed whether this was caused by mosaicism or chimerism.

There was no significant difference between the frequencies of aneuploidy in diploid androgenetic heterozygous conceptuses and triploid diandric heterozygous conceptuses, 11.5% and 14.2%, respectively (p = 0.813, Fisher’s exact test).

The mean age of women with diploid androgenetic heterozygous conceptuses, 33.1 years, was significantly higher than the mean age of women with diploid androgenetic homozygous conceptuses, 30.1 years (p = 0.012, Welch’s t-test), and significantly higher than the mean age of women with triploid diandric heterozygous conceptuses, 29.8 years (p = 0.004, Welch’s t-test) (Table 4).

**Table 4:** Age of women with conceptuses evacuated between 1986 and 2021 that were classified as diploid androgenetic homozygous, diploid androgenetic heterozygous, and triploid diandric heterozygous.

Age interval/years	Diploid androgenetic homozygous	Diploid androgenetic heterozygous	Triploid diandric heterozygous
< 25	53 (23.5%)	4 (7.7%)	13 (9.2%)
25–34	125 (55.3%)	28 (53.8%)	105 (74.5%)
35–44	32 (14.2%)	17 (32.7%)	23 (16.3%)
> 44	16 (7.1%)	3 (5.8%)	0 (0%)
Total	226	52	141
Mean age	30.1 years (SD 8.4, CI [29.0–31.2])	33.1 years (SD 7.5, CI [31.0–35.2])	29.8 years (SD 4.6, CI [29.0–30.5])

This tendency does not seem to be explained by time trend (Supplementary tables 1 and 2).

There was no significant difference between the mean age of women with aneuploid diploid androgenetic heterozygous conceptuses, 33.3 years, and the mean age of women with euploid diploid androgenetic heterozygous conceptuses, 33.1 years (p = 0.920, Welch’s t-test). Similarly, there was no significant difference between the mean age of women with aneuploid triploid diandric heterozygous conceptuses, 29.0 years, and the mean age of women with euploid triploid diandric heterozygous conceptuses, 29.9 years (p = 0.466, Welch’s t-test) (Supplementary table 3).

## Discussion

Among 278 diploid androgenetic conceptuses, we observed heterozygosity in 18.7%. In previous studies the observed frequency of heterozygosity in diploid androgenetic conceptuses has been reported to vary between 9.5 and 23% (Supplementary table 4^5,7,11–17^). However, some studies included relatively few cases. In addition, when a limited number of loci are analyzed, a heterozygous conceptus can be misclassified as homozygous. By analyzing three unlinked loci for which the father was heterozygous, and a locus specific for the Y chromosome, Fisher et al. observed heterozygosity in 8/35 diploid androgenetic conceptuses (23%)^13^. However, after correcting for the probability of homozygosity in all four loci in a dispermic androgenetic conceptus, the estimated frequency of heterozygosity rose to 25%. With several highly polymorphic loci analyzed, the risk of misclassification is reduced, and accordingly, we estimated the total number of heterozygous conceptuses misclassified as homozygous, to be 0.7, using a worst-case scenario. (Supplementary tables 5, 6, and 7).

The observed frequency of aneuploidy in diploid androgenetic heterozygous conceptuses was significantly higher than in the homozygous conceptuses, similarly to what was found by Usui et al. and Finley et al.^5,7^. We observed aneuploidy in 11.5% of diploid androgenetic heterozygous conceptuses, whereas Usui et al. and Finley et al. observed aneuploidy in 29% and 21%, respectively (Supplementary table 8). The fact that we observed a lower frequency is likely explained by that we identified aneuploidy by karyotyping, whereas Usui et al. and Finley et al. used SNP array analysis. Karyotyping only disclose the genetic constitution in cells that divide in vitro and possibly aneuploid cells are less likely to grow in vitro. Furthermore, in case of mosaicism, the karyotype of one cell line can be overlooked as only few cells are analyzed.

In general, aneuploidy is observed more frequently in conceptuses in mothers with high age^18^. In our study, the mean age of women with diploid androgenetic heterozygous conceptuses was significantly higher than the mean age of women with diploid androgenetic homozygous conceptuses. However, this was neither observed by Usui et al. nor by Zheng et al.^17,19^(Supplementary table 9). Furthermore, we found no significant difference between the mean age of women with aneuploid diploid androgenetic heterozygous conceptuses and the mean age of women with euploid diploid androgenetic heterozygous conceptuses (Supplementary table 3).

Traditionally, the mechanism behind a diploid androgenetic conceptus has been suggested to be fertilization of an empty oocyte by one or two spermatozoa. That this can be the case, is supported by recent observations in a mouse model^20^. Alternatively, maternal chromosomes may be lost after fertilization e.g. by a heterogoneic division^21,22^. This is supported by the observation of human mosaics where one spermatozoon has contributed to both a biparental and an androgenetic cell line^10^, and by the presence of additional chromosomes of maternal origin in otherwise androgenetic conceptus^5,23^.

In most diploid androgenetic heterozygous conceptuses, the paternally inherited sets of genomes likely originate from two spermatozoa^24^. A spermatozoon provides one pair of centrioles to the zygote, and an oocyte fertilized by two spermatozoa likely has two pairs of centrioles. One explanation for the high frequency of aneuploidy in heterozygous androgenetic conceptuses, could be that two pairs of centrioles in one zygote disturb the segregation of chromosomes, either directly (in case of fertilization of an anucleated oocyte), or indirectly because of a heterogoneic division (in case of fertilization a nucleated oocyte).

In almost all triploid diandric conceptuses, we identified heterozygosity for the paternally inherited sets of genomes, as also reported by others^3,14,16^. Using karyotyping, aneuploidy has been observed in 5.3%–20% of triploid diandric conceptuses^3,25–27^(Supplementary table 10). We found aneuploidy in 14.2% of the triploid diandric heterozygous conceptuses. The highest frequencies of aneuploidy were observed by Vejerslev et al. (20%) and us, which may be explained by that only Vejerslev et al. and we report mosaics^27^.

As for the diploids, it is unlikely that the high frequency of aneuploidy is explained by age, as we found no significant difference between the mean age of women with aneuploid triploid diandric heterozygous conceptuses and the mean age of women with euploid triploid diandric heterozygous conceptuses (Supplementary table 3).

Aneuploidy has also been observed in triploid digynic conceptuses^3,25,26^ (Supplementary table 10). Thus, one could speculate that in triploids, the high frequency of aneuploidy is caused by an increased risk of mitotic errors related to the abnormal number of chromosomes. However, as abnormal segregation in the oocyte meiosis is the cause of triploid digynic conceptuses, and dispermy is the most common cause of triploid diandric conceptuses^3^, it is also possible that aneuploidy in triploid digynic conceptuses is caused by the abnormal segregation in the oocyte meiosis, whereas aneuploidy in triploid diandric conceptuses is caused by the presence of two pairs of centrioles.

Among the limitations are that samples were collected, and analyses were conducted over a 37-year period, and that we did not systematically assess the parental origin of missing or surplus chromosomes. Furthermore, we did not review the histopathological diagnoses. However, molar pregnancies could be overlooked using histopathology^28^, and substantial interobserver and intraobserver variability in the morphological diagnosis of hydatidiform mole have been observed^29^. Thus, restricting the analysis to conceptuses classified as hydatidiform mole from morphological criteria, could have caused bias.

We could have misclassified heterozygous conceptuses as homozygous conceptuses, as we in most cases attempted to analyze 16 loci, only. However, the loci were unlinked, 15 loci were highly polymorphic, and we only included a conceptus if at least nine loci were successfully analyzed. Accordingly, the estimated number of misclassified heterozygous conceptuses was less than one.

Identifying aneuploidy by karyotyping may both lead to overestimation and underestimation of the frequency of aneuploidy. Aneuploidy may arise during cultivation whereas triploid and aneuploid cells may divide less frequently. The latter may explain that we observed aneuploidy less frequently than Usui et al., who used DNA analyses^5^. However, for the observations in triploid conceptuses, where we compared our results to the results of others using karyotyping, using karyotyping is unlikely to have influenced our conclusions significantly. Furthermore, karyotyping is advantageous for identification of triploid conceptuses, as the results of DNA marker analysis for a triploid diandric conceptus and a diploid mosaic conceptus with an androgenetic and biparental cell line may be identical^10^.

## Conclusion

Aneuploidy was observed frequently in heterozygous conceptuses—both in diploid androgenetic heterozygous conceptuses and in triploid diandric heterozygous conceptuses, in which the paternally inherited sets of genomes likely originate in two spermatozoa. Two pairs of centrioles in one zygote may disturb the segregation of chromosomes, causing aneuploidy.

### Supplementary Information

Supplementary Information.

## Data Availability

The datasets generated and/or analyzed during the current study are available from the corresponding author on reasonable request.
