# Supplementary material for: Aneuploidy is frequent in heterozygous diploid and triploid hydatidiform moles
Source: Sci Rep. 2024 Mar 22;14:6876. doi: 10.1038/s41598-024-57465-5 (PMC10960034; doi:10.1038/s41598-024-57465-5)
Supplement: Supplementary file 1 — Supplementary Information. [file 41598_2024_57465_MOESM1_ESM.docx]

# ANEUPLOIDY IS FREQUENT IN HETEROZYGOUS DIPLOID AND TRIPLOID HYDATIDIFORM MOLES

*P.Walbum^1^, L. Andreasen^2^, M. Geilswijk^2^, I. Niemann^3^, L. Sunde^1^

# SUPPLEMENTARY MATERIAL

Supplementary table 1. Age of women with conceptuses evacuated between 1986 and 2003 that were classified as diploid androgenetic homozygous, diploid androgenetic heterozygous, and triploid diandric heterozygous.

| Age interval/years | Diploid androgenetic homozygous | Diploid androgenetic heterozygous | Triploid diandric heterozygous |
| --- | --- | --- | --- |
| <25 | 31 (27.2%) | 2 (8.7%) | 11 (13.8%) |
| 25-34 | 66 (57.9%) | 16 (69.6%) | 59 (73.8%) |
| 35-44 | 12 (10.5%) | 3 (13.0%) | 10 (12.5%) |
| >44 | 5 (4.4%) | 2 (8.7%) | 0 (0%) |
| Total | 114 | 23 | 80 |
|  |  |  |  |
| Mean age | 28.8 years (SD 7.8, CI [27.3 – 30.2]) | 31.5 years (SD 7.9, CI [28.1 – 34.9]) | 28.6 years (SD 4.5, CI [27.6 – 29.6]) |

Supplementary table 2. Age of women with conceptuses evacuated between 2004 and 2021 that were classified as diploid androgenetic homozygous, diploid androgenetic heterozygous, and triploid diandric heterozygous.

| Age interval/years | Diploid androgenetic homozygous | Diploid androgenetic heterozygous | Triploid diandric heterozygous |
| --- | --- | --- | --- |
| <25 | 22 (19.6%) | 2 (6.9%) | 2 (3.3%) |
| 25-34 | 59 (52.7%) | 12 (41.4%) | 46 (75.4%) |
| 35-44 | 20 (17.9%) | 14 (48.3%) | 13 (21.3%) |
| >44 | 11 (9.8%) | 1 (3.4%) | 0 (0%) |
| Total | 112 | 29 | 61 |
|  |  |  |  |
| Mean age | 31.4 years (SD 8.9, CI 29.8 – 33.1]) | 34.4 years (SD 7.0, CI 31.8 – 37.1]) | 31.4 years (SD 4.1, CI [30.3 – 32.5]) |

Supplementary table 3. The mean age of women with aneuploid and euploid heterozygous conceptuses.

|  | Aneuploid heterozygous conceptuses | Euploid heterozygous conceptuses |
| --- | --- | --- |
| Diploid androgenetic | 33.3 years (SD 4.5; CI 28.6 - 38.1) (n=6) | 33.1 years (SD 7.8; CI 30.8 - 35.4) (n=46) |
| Triploid diandric | 29.0 years (SD 5.6; CI 26.3 - 31.6) (n=20) | 29.9 years (SD 4.4; CI 29.1 - 30.7) (n=121) |

Supplementary table 4. Studies where a frequency of heterozygosity among diploid androgenetic conceptuses, was reported.

| Study | Number of diploid androgenetic conceptuses | Frequency of heterozygosity | Method(s) used |
| --- | --- | --- | --- |
| Lawler et al. [1] | 20 | 10% (observed) | RFLP analysis of 5 loci for which the father was heterozygous |
| Fisher et al. [2] | 35 | 25% (estimated) | Minisatellite analysis of 3 loci and a locus specific for the Y chromosome, for which the father was heterozygous |
| Baasanjav et al. [3] | 27 | 11.1% (observed) | STR analysis: PowerPlex 16HS (16 loci) |
| Banet et al. [4] | 106 | 15.1% (observed) | STR analysis: AmpFlSTR Profiler (9 loci), and AmpFlSTR Identifiler (15 loci) |
| Khawajkie et al. [5] | 126 | 9.5% (observed) | STR analysis: PowerPlex 16HS (16 loci) |
| Zheng et al. [6] | 165 | 16.4% (observed) | STR analysis: PowerPlex 16HS (16 loci) |
| Usui et al. [7] ^a^ | 269 | 12.3% (observed) | STR analysis: PowerPlex 16HS (16 loci) |
| Xing et al. [9] | 144 | 15.3% (observed) | STR analysis: AmpFlSTR Profiler (9 loci), and AmpFlSTR Identifiler (15 loci) |
| Finley et al. [10] | 196 | 9.7% (observed) | Genome wide SNP analysis |
| Present study | 278 | 18,7% (estimated)^b^ | STR analysis: AmpFlSTR Identifiler (15 loci), or NGM SElect™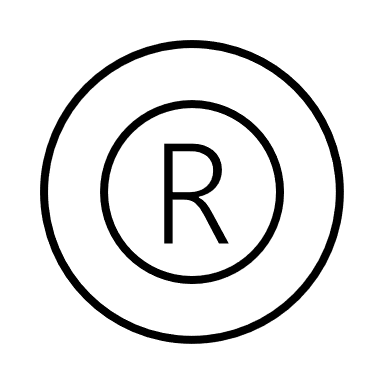 (16 loci) |

^a^ Some of the cases were previously published in [8], where the frequency of heterozygosity was 12.1%, among 232 diploid androgenetic conceptuses.

^b^ We observed 52 conceptuses with aneuploidy among 278 conceptuses = 18.71%. We calculated that 0.04 conceptuses could have been misclassified as homozygous (Supplementary table 6) and estimated 52.04 conceptuses had aneuploidy among 278 conceptuses = 18.72%.

Supplementary table 5. The frequencies of heterozygosity in loci analyzed with the AmpFlSTR Identifiler kit and the AmpFlSTR NGM SElect™
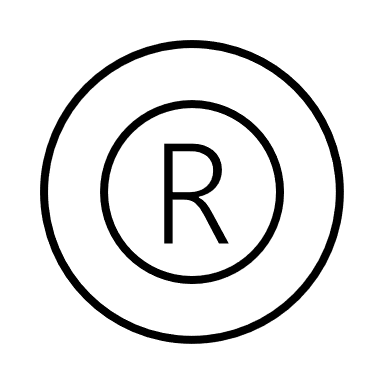
 kit, using observations of heterozygosity in the Scandinavian populations.

| Kit | Mean | Range |
| --- | --- | --- |
| AmpFlSTR Identifiler kit ^a^ | 78.2% | 53.4% - 87.2% |
| AmpFlSTR NGM SElect™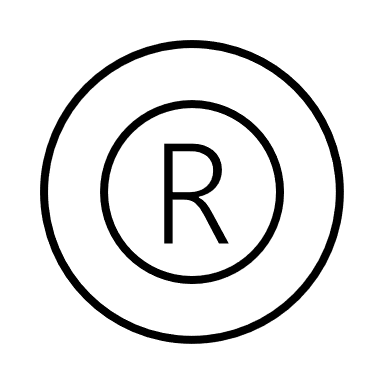 kit ^b^ | 82.5% | 73.4% - 94.8% |

^a^ [11]

^b^ [12]

Supplementary table 6. The estimated number of diploid androgenetic heterozygous conceptuses misclassified as homozygous, using a mean frequency of heterozygosity of 80% in autosomal loci.

| Number of loci analyzed | Number of diploid androgenetic conceptuses classified as homozygous | Estimated number of diploid androgenetic conceptuses misclassified as homozygous ^a^ |
| --- | --- | --- |
| 9 | 1 | 0.6^8^ x 0.33^1^ x 1 = 0.0055 |
| 10 | 1 | 0.6^9^ x 0.33^1^ x 1 = 0.0033 |
| 12 | 1 | 0.6^11^ x 0.33^1^ x 1 = 0.0012 |
| 14 | 1 | 0.6^13^ x 0.33^1^ x 1 = 0.0004 |
| 15 | 6 | 0.6^14^ x 0.33^1^ x 6 = 0.0016 |
| 16 | 173 | 0.6^15^ x 0.33^1^ x 173 = 0.0268 |
| At least 17 | 43 | 0.6^16^ x 0.33^1^ x 43 = 0.0040 |
| Total | 226 | 0.0428 |

^a^ Assuming a mean frequency of heterozygosity for autosomal loci of 80% (Supplementary table 6), the probability of a “father” being homozygous in a given autosomal locus, is 20%. For loci where the “father” is homozygous, a dispermic conceptus will be homozygous. For the 80% of “fathers” being heterozygous in a given locus, the probability that a dispermic conceptus is homozygous in the locus, is 50%. Thus, the probability that a dispermic conceptus is homozygous in a given autosomal locus is (80%x50%) + 20% = 60%. For the sex chromosomes, the probability that a dispermic conceptus is homozygous is 33%, as diploid androgenetic conceptuses with two Y chromosomes are not seen [13]. The estimated number of diploid conceptuses classified as homozygous that were misclassified = (The probability of homozygosity in one polymorphic autosomal locus in a heterozygous conceptus ^the number of polymorphic autosomal loci analyzed) x the frequency of homozygosity for the sex chromosome in a heterozygous conceptus x the number of conceptuses classified as homozygous.

Supplementary table 7. The estimated number of diploid androgenetic heterozygous conceptuses misclassified as homozygous, using a mean frequency of heterozygosity of 53.4% in autosomal loci (the lowest frequency of heterozygosity for the autosomal loci analyzed in the AmpFlSTR Identifiler kit).

| Number of loci analyzed | Number of diploid androgenetic conceptuses classified as homozygous | Estimated number of diploid androgenetic conceptuses misclassified as homozygous ^a^ |
| --- | --- | --- |
| 9 | 1 | 0.733^8^ x 0.33^1^ x 1 = 0.0275 |
| 10 | 1 | 0.733^9^ x 0.33^1^ x 1 = 0.0202 |
| 12 | 1 | 0.733^11^ x 0.33^1^ x 1 = 0.0108 |
| 14 | 1 | 0.733^13^ x 0.33^1^ x 1 = 0.0058 |
| 15 | 6 | 0.733^14^ x 0.33^1^ x 6 = 0.0256 |
| 16 | 173 | 0.733^15^ x 0.33^1^ x 173 = 0.5409 |
| At least 17 | 43 | 0.733^16^ x 0.33^1^ x 43 = 0.0985 |
| Total | 226 | 0.729 |

^a^ Assuming a mean frequency of heterozygosity for autosomal loci of 53.4%, the probability of a “father” being homozygous in a given autosomal locus, is 46.6%. For loci where the “father” is homozygous, a dispermic conceptus will be homozygous. For the 53.4% of “fathers” being heterozygous in a given locus, the probability that a dispermic conceptus is homozygous in the locus, is 50%. Thus, the probability that a dispermic conceptus is homozygous in a given autosomal locus is (53.4%x50%) + 46.6% = 73.3%. For the sex chromosomes, the probability that a dispermic conceptus is homozygous is 33%, as diploid androgenetic conceptuses with two Y chromosomes are not seen [13]. The estimated number of diploid conceptuses classified as homozygous that were misclassified = (The probability of homozygosity in one polymorphic autosomal locus in a heterozygous conceptus ^the number of polymorphic autosomal loci analyzed) x the frequency of homozygosity for the sex chromosome in a heterozygous conceptus x the number of conceptuses classified as homozygous.

Supplementary table 8. Comparison between the two studies that observed aneuploidy in diploid androgenetic conceptuses, stratified by genotype.

|  | Usui et al. [7] | Finley et al. [10] | Present study |
| --- | --- | --- | --- |
| Study population | Conceptuses suspected to be molar pregnancy based on ultrasonographic findings, macroscopic analysis of the specimen, or histopathologic analyses of the specimen.  238 homozygous HMs and 31 heterozygous HMs were identified. | Clinical products of conception samples referred to a commercial laboratory (INVITAE) for SNP array analyses.  177 cases with whole-genome uniparental isodisomy and 19 cases with whole-genome uniparental heterodisomy, were identified. | Conceptuses suspected to be molar pregnancy based on ultrasonographic findings, macroscopic inspection of specimen or hormone level.  226 diploid androgenetic homozygous conceptuses and 52 diploid androgenetic heterozygous conceptuses were identified. |
| Time period and location | Samples were collected between 2007 and 2018 at Chiba University Hospital and affiliated clinics and hospitals in Japan. | Multiyears study | Samples were received between 1986 and 2022 from hospitals in western Denmark. |
| Method(s) | STR analysis and  SNP array analysis | SNP array analysis | STR analysis and karyotyping |
| Frequency of aneuploidy in homozygous diploid androgenetic conceptuses | 0/238 (0%) | 1/177 (0.6%) | 1/226 (0.4%) |
| Frequency of aneuploidy in heterozygous diploid androgenetic conceptuses | 9/31 (29.0%) | 4/19 (21%) | 6/52 (11.5%) |

Supplementary table 9. Comparison between the three studies that observed mean age of women with a homozygous or a heterozygous diploid androgenetic conceptus.

|  | Usui et al. [8] | Zheng et al. [6] | Present study |
| --- | --- | --- | --- |
| Study population | Conceptuses suspected to be molar pregnancy based on ultrasonographic findings or macroscopic analysis of specimen or histopathologic analyses of specimen.  204 homozygous HMs and 28 heterozygous HMs, were identified. | Conceptuses with histological features suspicious for early complete mole, fully developed complete mole or partial mole.  138 homozygous HMs and 27 heterozygous HMs were identified. | Conceptuses with clinical and/or histological features suspicious for molar pregnancy.  231 diploid androgenetic homozygous conceptuses and 52 diploid androgenetic heterozygous conceptuses, were identified. |
| Time period and locations | Samples were collected between 2007 and 2017 at Chiba University Hospital and affiliated clinics and hospitals in Japan. | Samples were collected between 2015 and 2019 at Beijing Obstetrics and Gynecology Hospital. | Samples were received between 1986 and 2022 from hospitals in western Denmark. |
| Mean age of women with a homozygous diploid conceptus (years) | 32.5 (SD: 7.9) | 31.6 | 30.0 (SD: 8.4) |
| Mean age of women with a heterozygous diploid conceptus (years) | 32.1 (SD: 6.8) | 29.3 | 33.1 (SD: 7.5) |

Supplementary table 10. Comparison between the studies that used karyotyping and observed a frequency of aneuploidy in diandric and/or digynic triploid conceptuses.

| Study | Frequency of aneuploidy in triploid diandric conceptuses | Frequency of aneuploidy in triploid digynic conceptuses |
| --- | --- | --- |
| Jacobs et al. [14] | Among 57 conceptuses aneuploidy was observed in three (5.3%). | Among 22 conceptuses, aneuploidy was observed in two (9.1%). |
| Uchida & Freeman [15] | Among 52 triploid diandric conceptuses, aneuploidy was observed in three (5.8%). | Among 29 triploid digynic conceptuses, aneuploidy was observed in four (13.8%). |
| Zaragoza et al. [16] | Among 60 triploid diandric conceptuses, aneuploidy was observed in eight (13.3%). | Among 27 triploid digynic conceptuses, aneuploidy was observed in two (7.4%). |
| Vejerslev et al. [17] | Among 25 triploid diandric conceptuses, aneuploidy was observed in five (20 %). Two of these were with one euploid and one aneuploid cell line. |  |
| Present study | Among 142 triploid diandric conceptuses, aneuploidy was observed in 21 (14.8%). Nine of these were with one euploid and one aneuploid cell line. |  |

# REFERENCES

[1] S. D. Lawler, V. J. Pickthall, R. A. Fisher, S. Povey, M. W. Evans, and Szulman A. E., “Genetic studies of complete and partial hydatidiform moles,” *The Lancet*, pp. 580–580, 1979, doi: 10.1016/s0140-6736(79)91632-5.

[2] R. A. Fisher, S. Povey, A. J. Jeffreys, C. A. Martin, I. Patel, and S. D. Lawler, “Frequency of heterozygous complete hydatidiform moles, estimated by locus-specific minisatellite and Y chromosome-specific probes,” *Hum Genet*, vol. 82, pp. 259–263, 1989, doi: 10.1007/BF00291166.

[3] B. Baasanjav *et al.*, “The risk of post-molar gestational trophoblastic neoplasia is higher in heterozygous than in homozygous complete hydatidiform moles,” *Human Reproduction*, vol. 25, no. 5, pp. 1183–1191, 2010, doi: 10.1093/humrep/deq052.

[4] N. Banet *et al.*, “Characteristics of hydatidiform moles: Analysis of a prospective series with p57 immunohistochemistry and molecular genotyping,” *Modern Pathology*, vol. 27, no. 2, pp. 238–254, Feb. 2014, doi: 10.1038/modpathol.2013.143.

[5] Y. Khawajkie *et al.*, “Comprehensive analysis of 204 sporadic hydatidiform moles: revisiting risk factors and their correlations with the molar genotypes,” *Modern Pathology*, vol. 33, no. 5, pp. 880–892, May 2020, doi: 10.1038/s41379-019-0432-4.

[6] X. Z. Zheng *et al.*, “Heterozygous/dispermic complete mole confers a significantly higher risk for post-molar gestational trophoblastic disease,” *Modern Pathology*, vol. 33, no. 10, pp. 1979–1988, Oct. 2020, doi: 10.1038/s41379-020-0566-4.

[7] H. Usui, A. Sato, and M. Shozu, “Parental contribution to trisomy in heterozygous androgenetic complete moles,” *Sci Rep*, vol. 10, no. 1, Dec. 2020, doi: 10.1038/s41598-020-74375-4.

[8] H. Usui *et al.*, “Gestational Trophoblastic Neoplasia from Genetically Confirmed Hydatidiform Moles: Prospective Observational Cohort Study,” *International Journal of Gynecological Cancer*, vol. 28, no. 9, pp. 1772–1780, Nov. 2018, doi: 10.1097/IGC.0000000000001374.

[9] D. Xing, E. Adams, J. Huang, and B. M. Ronnett, “Refined diagnosis of hydatidiform moles with p57 immunohistochemistry and molecular genotyping: updated analysis of a prospective series of 2217 cases,” *Modern Pathology*, vol. 34, no. 5, pp. 961–982, May 2021, doi: 10.1038/s41379-020-00691-9.

[10] J. Finley *et al.*, “The genomic basis of sporadic and recurrent pregnancy loss: a comprehensive in-depth analysis of 24,900 miscarriages,” 2022, doi: 10.1016/j.

[11] K. Montelius, A. O. Karlsson, and G. Holmlund, “STR data for the AmpFℓSTR Identifiler loci from Swedish population in comparison to European, as well as with non-European population,” *Forensic Sci Int Genet*, vol. 2, no. 3, Jun. 2008, doi: 10.1016/j.fsigen.2007.12.005.

[12] C. Tomas, H. S. Mogensen, S. L. Friis, C. Hallenberg, M. C. Stene, and N. Morling, “Concordance study and population frequencies for 16 autosomal STRs analyzed with PowerPlex® ESI 17 and AmplSTR® NGM SElect^TM^ in Somalis, Danes and Greenlanders,” *Forensic Sci Int Genet*, vol. 11, no. 1, 2014, doi: 10.1016/j.fsigen.2014.04.004.

[13] Ohama K *et al.*, “Dispermic origin of XY hydatidiform moles,” *Nature*, vol. 292, pp. 551–552, 1981, doi: 10.1038/292551a0.

[14] P. A. Jacobs, A. E. Szulman, J. Funkhouser, J. S. Matsuura, and C. C. Wilson, “Human triploidy: relationship between parental origin of the additional haploid complement and development of partial hydatidiform mole,” *Ann Hum Genet*, vol. 46, no. 3, pp. 223–231, 1982, doi: 10.1111/j.1469-1809.1982.tb00714.x.

[15] Uchida Irene A. and Freeman Viola C. P., “Triploidy and chromosomes,” *Am J Obstet Gynecol*, vol. 151, no. 1, pp. 65–69, 1985, doi: 10.1016/0002-9378(85)90426-0.

[16] M. V Zaragoza, U. Surti, R. W. Redline, E. Millie, A. Chakravarti, and T. J. Hassold, “Parental Origin and Phenotype of Triploidy in Spontaneous Abortions: Predominance of Diandry and Association with the Partial Hydatidiform Mole,” *Am. J. Hum. Genet*, vol. 66, pp. 1807–1820, 2000, doi: 10.1086/302951.

[17] L. O. Vejerslev, J. Dissing, H. E. Hansen, and H. Poulsen, “Hydatidiform mole: genetic origin in polyploid conceptuses,” *Hum Genet*, vol. 76, pp. 11–19, 1987, doi: 10.1007/BF00283043.
